# Whole Blood Metabolomic Profiling of Mice with Tacrolimus-Induced Chronic Nephrotoxicity: NAD^+^ Depletion with Salvage Pathway Impairment

**DOI:** 10.3390/antiox14010062

**Published:** 2025-01-07

**Authors:** Sho Nishida, Tamaki Ishima, Daiki Iwami, Ryozo Nagai, Kenichi Aizawa

**Affiliations:** 1 Division of Clinical Pharmacology, Department of Pharmacology, Jichi Medical University, Shimotsuke 329-0498, Japan; 2 Division of Renal Surgery and Transplantation, Department of Urology, Jichi Medical University, Shimotsuke 329-0498, Japan; 3 Jichi Medical University, Shimotsuke 329-0498, Japan; 4 Clinical Pharmacology Center, Jichi Medical University Hospital, Shimotsuke 329-0498, Japan; 5 Division of Translational Research, Clinical Research Center, Jichi Medical University Hospital, Shimotsuke 329-0498, Japan

**Keywords:** tacrolimus (TAC), TAC nephrotoxicity, NAD^+^, metabolomics, chronic kidney disease biomarkers, ADMA, SDMA, 5′-methyl-2′-deoxycytidine, threo-β-Methylaspartic acid, H-Asp (Gly-OH)-OH, CKD biomarker

## Abstract

Tacrolimus (TAC)-induced chronic nephrotoxicity (TAC nephrotoxicity) is a serious issue for long-term graft survival in kidney transplantation. However, the pathophysiology of TAC nephrotoxicity remains unclear. In this study, we analyzed whole blood samples from mice that developed TAC nephrotoxicity in order to discover its mechanism. Mice were divided into a TAC group and a control group (*n* = 5 per group). The TAC group received TAC subcutaneously (1 mg/kg/day for 28 days), while the control group received normal saline instead. After the administration period, whole blood was collected and metabolomic analysis was performed, revealing significant changes in 56 metabolites. The major metabolic changes were related to uremic toxins, vascular damage, and NAD^+^. NAD^+^ levels were significantly lower in the TAC group, and ADP-ribose, nicotinamide, and nicotinamide N-oxide, which are degradation products of NAD^+^, were significantly higher, suggesting impairment of the NAD^+^ salvage pathway. NAD^+^ deficiency suggests cellular aging and mitochondrial dysfunction, which may induce vascular damage and chronic kidney disease. Our study demonstrated a correlation between low NAD^+^ levels and the pathophysiology of TAC nephrotoxicity.

## 1. Introduction

Tacrolimus (TAC) is a key immunosuppressive drug that has improved short-term kidney graft survival by preventing acute rejection in kidney transplantation [1]. Approximately 90% of immunosuppressive regimens worldwide are managed with two drugs: TAC and Mycophenolate mofetil [2]. On the other hand, chronic nephrotoxicity caused by TAC, known as TAC-induced chronic nephrotoxicity (TAC nephrotoxicity), is a significant barrier to long-term graft survival [3]. TAC nephrotoxicity is defined as an irreversible and progressive form of chronic kidney damage caused by continuous exposure to TAC [4]. Within 10 years of kidney transplantation, the incidence of chronic kidney damage due to prolonged exposure to TAC is estimated at around 70% of all kidney recipients [5]. Although TAC nephrotoxicity is thought to be involved in damage to vascular endothelium and proximal tubules [6], its detailed pathophysiology remains unclear, and there are no effective treatments thus far.

A definitive diagnosis of TAC nephrotoxicity can only be made through pathological findings from the transplanted kidney graft via needle or open surgical biopsy [3]. Characteristic pathological findings of TAC nephrotoxicity include specific arterial hyalinosis and interstitial fibrosis [6,7]. By the time a definitive diagnosis is made via graft biopsy, irreversible changes have often already occurred, preventing intervention. Therefore, it is crucial to assess the disease’s pathophysiology using less invasive techniques and to evaluate the condition at an earlier stage.

With technical advances in mass spectrometry in recent years, many proteins, enzymes, and metabolites can be detected more precisely in small samples [8,9,10]. This technique has been adapted for metabolomics, and comprehensive analysis has been used to understand mechanisms of various diseases. In chronic kidney diseases (CKDs), metabolomic analysis focused on blood samples is being used to investigate pathophysiology and to search for new biomarkers [11,12,13,14]. However, there have been no reports of metabolomic analysis using whole blood samples about TAC chronic nephrotoxicity. As it would be beneficial to know the mechanism of TAC nephrotoxicity and to assay biomarkers by using easily obtainable blood samples, we comprehensively analyzed metabolomic changes in whole blood to evaluate the pathophysiology of TAC nephrotoxicity.

## 2. Materials and Methods

### 2.1. Sample Collection

The TAC nephrotoxicity mouse model was created based on a previously reported method [15,16]. The sample size was determined based on previous studies employing similar models [15,17,18,19]. In brief, male 7-week-old ICR mice were housed in a mouse cage (Maxi-Miser^®^ Caging System #5, Oriental Giken Inc., Tokyo, Japan). Mice were kept on a 12/12 h light–dark cycle. Humidity was maintained at 40 ± 10%, and the temperature was kept at 23 ± 2 °C in the room. Mice were given free access to a low-sodium diet (0.01% sodium, CLEA Japan, Inc., Tokyo, Japan) and to tap water. After 7 days, mice were divided into two groups (n = 5 per group). The TAC group received continuous subcutaneous administration of TAC (Prograf^®^, Astellas Pharma, Inc., Tokyo, Japan) at 1 mg/kg/day using an osmotic pump (ALZET^®^ osmotic pump 2004, ALZET Osmotic pumps, Cupertino, CA, USA), while the control group received normal saline instead. After 28 days of continuous subcutaneous administration, samples were collected. Whole blood was collected from the inferior vena cava with a 26 G needle and a 1 mL syringe under isoflurane anesthesia (induction concentration 4–5%, maintenance concentration 2–3%). Samples were frozen and stored at −80 °C. This animal protocol was approved by the Institutional Animal Care and Concern Committee at Jichi Medical University (protocol code: 23008-01, 3 July 2023).

### 2.2. Metabolite Extraction

A quantity of 50 µL of whole blood was added to 200 µL of methanol containing internal standards (H3304-1002, Human Metabolome Technologies, Inc. (HMT), Tsuruoka, Japan). The extract was thoroughly mixed with 150 µL of Milli-Q water, after which 300 µL of the mixture was centrifugally filtered through a 5-kDa cutoff filter (ULTRAFREE MC PLHCC, HMT, Tsuruoka, Japan) at 9100× *g* and 4 °C to remove macromolecules. Filtrate was then evaporated to dryness under vacuum and reconstituted in 50 µL of Milli-Q water for metabolomic analysis.

### 2.3. Metabolomic Analysis

Metabolomic analysis was conducted with HMT’s ω Scan package, using capillary electrophoresis Fourier transform mass spectrometry (CE-FTMS) based on methods described previously [9]. Briefly, CE-FTMS analysis was carried out using an Agilent 7100 CE capillary electrophoresis system equipped with a Q Exactive Plus (Thermo Fisher Scientific Inc., Waltham, MA, USA), Agilent 1260 isocratic HPLC pump, Agilent G1603A CE-MS adapter kit, and Agilent G1607A CE-ESI-MS sprayer kit (Agilent Technologies, Inc., Santa Clara, CA, USA). The system was controlled by an Agilent MassHunter workstation, with LC/MS data acquisition software (ver B.08.00) for 6200 series TOF/6500 series Q-TOF (Agilent Technologies) and Xcalibur (Thermo Fisher Scientific). Metabolites were separated using a fused silica capillary (50 μm i.d. × 80 cm total length) with commercial electrophoresis buffer (H3301-1001 and I3302-1023 for cation and anion analyses, respectively, HMT) as the electrolyte. The spectrometer was scanned from *m*/*z* 60 to 900 in positive mode, and from *m*/*z* 70 to 1050 in negative mode. Peaks were extracted using MasterHands automatic integration software (ver2.19.0.2, Keio University, Tsuruoka, Japan) in order to obtain peak information, including *m*/*z*, peak area, and migration time (MT) [10]. Signal peaks corresponding to isotopomers, adduct ions, and other product ions of known metabolites were excluded, and the remaining peaks were annotated using HMT’s metabolite database based on their *m*/*z* values and MTs. Areas of annotated peaks were then normalized to internal standards and sample volume in order to obtain relative levels of each metabolite. Hierarchical cluster analysis (HCA) and principal component analysis (PCA) [20] were performed using MATLAB (ver.7.14.2, MathWorks, Inc., Natick, MA, USA) and R programs (R: A language and environment for statistical computing. R Foundation for Statistical Computing, version 3.5.2, Vienna, Austria. URL: https://www.R-project.org/, accessed on 31 May 2024). Detected metabolites were plotted on metabolic pathway maps using VANTED software (ver.2.1.0, University of Konstanz, Konstanz, Germany) [21].

### 2.4. Statistical Analysis and Graphic Design

Statistical analysis of metabolites measured by CE-FTMS was performed using Welch’s *t*-test in R 3.5.2, and figures were created with GraphPad Prism (ver.7.00, GraphPad Software, Boston, MA, USA). *p* < 0.05 was considered statistically significant.

## 3. Results

### 3.1. Metabolites in Whole Blood Samples and PCA/HCA

**Figure 1:**
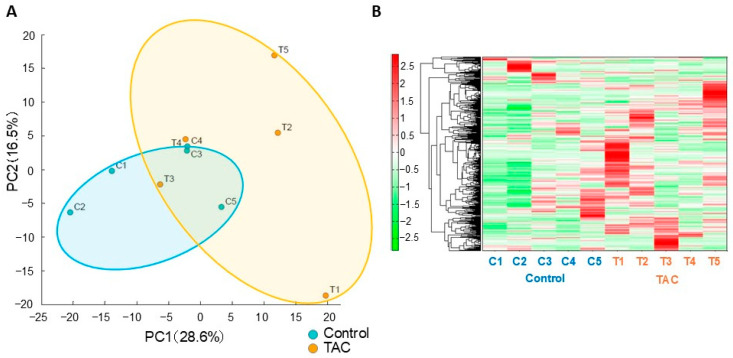
Principal component analysis and a heatmap of hierarchical cluster analysis. A total of 527 metabolites in whole blood were examined in our metabolomic analysis. Differences were found between the TAC and control groups using PCA and HCA. Control samples are numbered from C1 to C5, and TAC samples from T1 to T5 (*n* = 5/group). (**A**) shows results of PCA. The horizontal axis indicates the first principal component score (PC1), and the vertical axis shows the second (PC2). (**B**) presents a heatmap displaying HCA of metabolites with a tree of sequences. The bar on the right side of the heatmap shows the relationship between measured values and the color of each sample. Red indicates values higher than average, and green denotes values lower than average. The vertical axis shows metabolites. PCA: principal component analysis; HCA: heatmap displaying hierarchical clustering analysis.

A total of 527 metabolites were detected, and 56 of these showed significant changes after TAC administration (Figure 1A,B). PCA and HCA suggested different metabolic distributions between the TAC and control groups.

### 3.2. Metabolites with Significant Differences

Of the 56 metabolites with differences, 52 were significantly higher in the TAC group, while 4 were significantly lower (Table 1). Among these 56 metabolites, 11 metabolites showed significant changes in both whole blood and kidney tissue (Table 1) [15]. These 11 metabolites are as follows: threo-β-methlaspartic acid, γ-carboxyglutamic acid, ascorbate 2-sulfate, glucaric acid, 1-methl-4-imidazoleacetic acid, imidazolelactic acid, N-Acetyltaurine, 2′-deoxycytidine, uridine diphosphate-N-acetylglucosamine (UDP-N-acetylglucosamine), H-Aspartic (Glycine -OH)-OH ((H-Asp (Gly-OH)-OH), and creatine.

### 3.3. Main Metabolic Categories in Blood Samples

**Figure 2:**
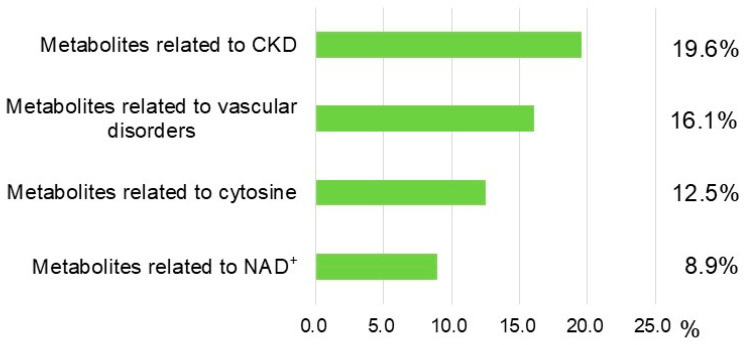
Categorization of metabolites with significant differences. A total of 56 metabolites differed significantly between the TAC and control groups in this metabolomic analysis. Statistical analysis was performed with Welch’s *t*-test. *p* < 0.05 was considered statistically significant. Horizontal green bars and numbers next to the bars show ratios of metabolite groups versus all metabolites with significant differences. Some metabolites categorized in each group were classified into multiple categories. Details of metabolites categorized in each group are presented in Supplemental Table S1. CKD: chronic kidney disease, NAD^+^: nicotinamide adenine dinucleotide.

Categories of metabolites in blood samples are shown in Figure 2. The main metabolite categories included are 11 metabolites related to uremic toxins (19.6%), 9 related to vascular damage (16.1%), 7 related to cytosine metabolism (12.5%), and 5 related to nicotinamide adenine dinucleotide (NAD^+^) metabolism (8.9%) (Figure 2). Several metabolites overlapped between these groups (Supplemental Table S1).

### 3.4. Metabolites Related to Uremic Toxins and CKD

**Figure 3:**
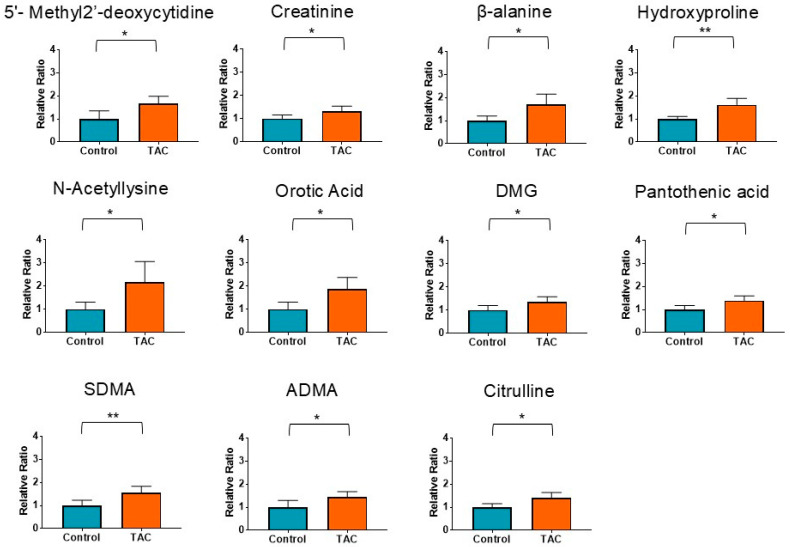
Metabolites related to uremic toxins and CKD are measured by metabolomic analysis. Orange bars represent the TAC group, and blue columns represent the Control group. The vertical axis is the relative ratio. The ratio was calculated as follows: the relative ratio = normalized ratio of Tac group/normalized ratio of control group. (* *p* < 0.05, ** *p* < 0.01) (*n* = 5/group). CKD: chronic kidney disease; DMG: N, N-Dimethylglycine; SDMA: symmetric dimethylarginine; ADMA: asymmetric dimethylarginine.

The main metabolite group was related to uremic toxins and CKD (Figure 3). A total of 11 metabolites related to uremic toxins and CKD were significantly higher in the TAC group. Among uremic toxins, symmetric dimethylarginine (SDMA) and asymmetric dimethylarginine (ADMA), which are derivatives of arginine, were significantly higher in the TAC group. This suggests that whole blood samples in the TAC group reflect the presence of CKD.

### 3.5. Metabolites Related to Vascular Disorders

**Figure 4:**
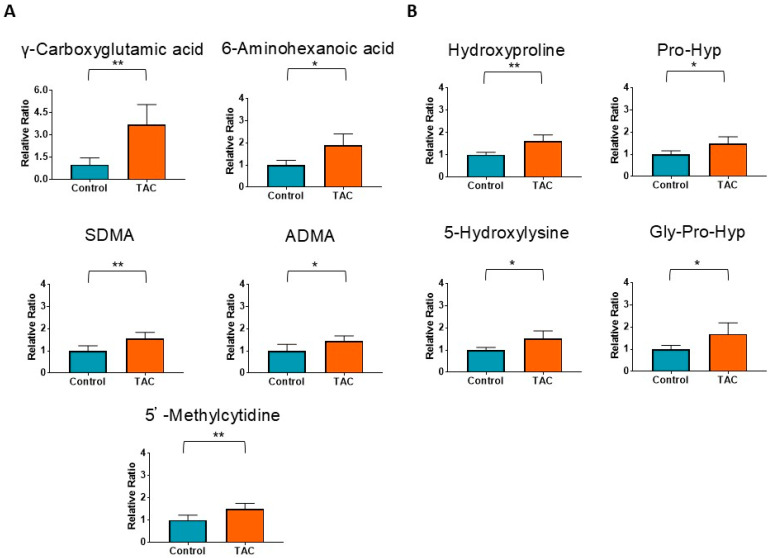
Metabolites related to vascular disorders measured by metabolomic analysis. Orange columns represent the TAC group, and blue columns represent the Control group. The vertical axis is the relative ratio. The ratio was calculated as follows: the relative ratio = normalized ratio of Tac group/normalized ratio of control group. (**A**) shows metabolites suggesting vascular damage. (**B**) shows metabolites as degradation products derived from collagen. (* *p* < 0.05, ** *p* < 0.01) (*n* = 5/group). DMG: N, N-Dimethylglycine; SDMA: symmetric dimethylarginine; ADMA: asymmetric dimethylarginine.

Nine metabolites related to vascular disorders are shown in Figure 4. Among them, five metabolites shown in Figure 4A are related to vascular calcification, thrombosis, and vascular damage. The remaining four metabolites are decomposition products of collagen and were significantly higher in the TAC group (Figure 4B).

### 3.6. Low NAD^+^ Levels and Salvage Pathway Impairment

**Figure 5:**
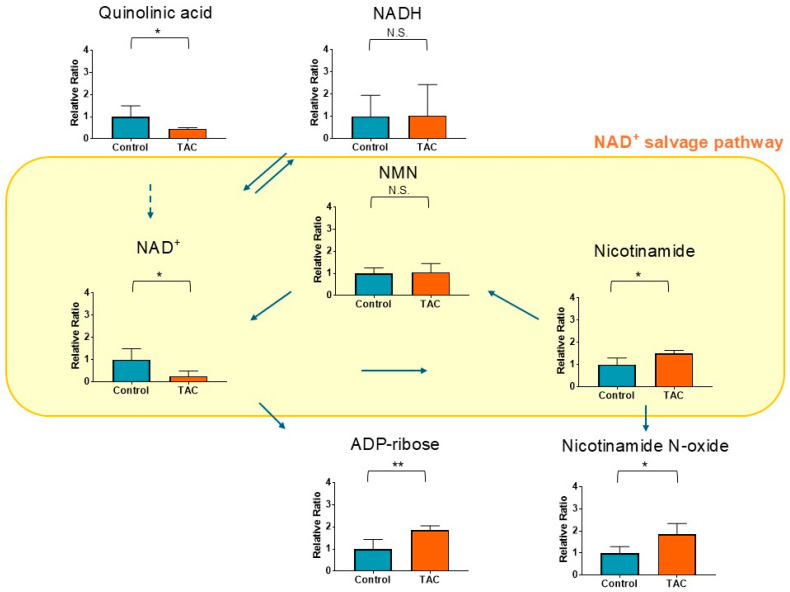
NAD^+^-related metabolites measured by metabolomic analysis. Orange columns represent the TAC group, and blue columns represent the Control group. The vertical axis is the relative ratio. The ratio was calculated as follows: the relative ratio = normalized ratio of Tac group/normalized ratio of control group. Solid arrows indicate relationships between metabolites and their precursors. A dotted arrow indicates an indirect relationship to metabolite production. The yellow area shows the NAD^+^ salvage pathway and its components. (* *p* < 0.05, ** *p* < 0.01) (*n* = 5/group). NAD^+^: Nicotinamide adenine dinucleotide; NMN: Nicotinamide mononucleotide; N.S.: not significant.

NAD^+^ was significantly reduced in the TAC group (Figure 5). Quinolinic acid, a precursor of NAD^+^, was also lower after TAC exposure. Although nicotinamide mononucleotide (NMN), a metabolite related to the NAD^+^ salvage pathway, showed no significant difference between the two groups, Nicotinamide (NAM) was significantly higher in the TAC group. In addition, Adenosine diphosphate-ribose (ADP-ribose) and Nicotinamide N-oxide (NNO), degradation products of NAD^+^, were significantly higher in the TAC group. These results suggest impairment of the NAD^+^ salvage pathway and disruption of the balance between synthesis and consumption of NAD^+^.

### 3.7. Metabolites Related to Cytosine and Its Aberrant Catabolites

**Figure 6:**
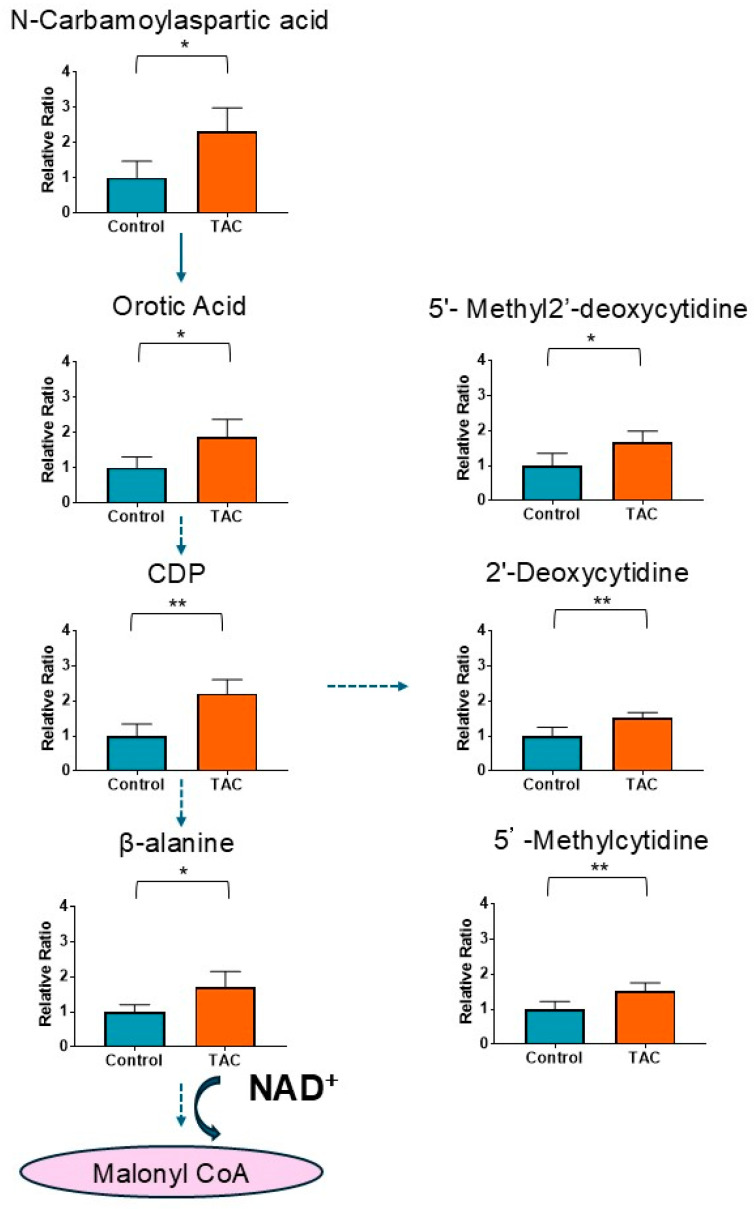
Cytosine-related metabolites measured by metabolome analysis. Orange columns represent the TAC group, and blue columns represent the Control group. The vertical axis is the relative ratio. The ratio was calculated as follows: the relative ratio = normalized ratio of Tac group/normalized ratio of control group. A Solid arrow indicates relationships between metabolites and their precursors. Dotted arrows indicate indirect relationships to metabolite production. Malonyl CoA was not detected. (* *p* < 0.05, ** *p* < 0.01) (*n* = 5/group). CDP: cytidine diphosphate; NAD^+^: Nicotinamide adenine dinucleotide.

Seven metabolites related to cytosine metabolism with significant difference are shown in Figure 6. All of them were significantly higher in the TAC group. Additionally, metabolites with a cytosine structure included Cytidine diphosphate (CDP), 2′-deoxycytidine, 5′-methyl-2′-deoxycytidine, and 5′-methylcytidine. Orotic acid, 5′-methyl-2′-deoxycytidine and β-alanine are related to CKD (Supplemental Table S1). In addition, as 5′-methylcytidine is also related to vascular calcification, 5′-methylcytidine belongs to both the cytosine group and the vascular disorders group (Supplemental Table S1).

## 4. Discussion

### 4.1. Pathophysiology and Potential Biomarkers for TAC Nephrotoxicity

To the best of our knowledge, this is the first study to examine TAC nephrotoxicity in whole blood samples. Whole blood contains high concentrations of metabolites related to antioxidation, energy metabolism, sugar phosphates, nucleotides, and nucleotide-sugar derivatives [22,23]. For TAC nephrotoxicity, previous studies have reported that the pathophysiology involves increased reactive oxygen species (ROS) production, decreased antioxidant function, and impaired energy metabolism [24,25,26,27]. In addition, reports on biomarkers for TAC nephrotoxicity have been so far limited to studies on acute kidney injury or analyses using urine samples [28,29]. Therefore, this metabolomic analysis using whole blood samples constitutes an effective way to evaluate the pathophysiology of TAC nephrotoxicity and to find potential new biomarkers for chronic kidney damage caused by TAC.

Among the 56 metabolites that differed between the two groups, most were significantly higher in the TAC group. Metabolites related to uremic toxins and vascular disorders suggest that whole blood samples reflect early changes related to TAC nephrotoxicity that are not fully captured in kidney tissue. Of the significant blood metabolites, 11 are also significantly changed in kidney tissues, according to previous reports on TAC nephrotoxicity (Table 1). In addition, these metabolites have not been reported in other CKD animal models or in metabolomic analyses of human samples, except for creatine [11,18,30,31]. Therefore, these metabolites could serve as specific biomarkers for TAC nephrotoxicity.

### 4.2. Metabolites Related to Uremic Toxins and CKD

In this study, 11 metabolites previously reported as uremic toxins or identified in CKD cases were detected [11,13,31,32,33,34]. Notably, SDMA and ADMA, which are derived from arginine [35], were both significantly higher in the TAC group. SDMA and ADMA are toxic, non-proteinogenic amino acids that suppress nitric oxide (NO) production in vascular walls, leading to vascular damage [35,36,37]. More than 90% of SDMA is excreted through the kidneys, and the increase of SDMA in blood indicates kidney damage [38]. ADMA is degraded by dimethylarginine dimethlamiohydrolase into citrulline and dimethylamine, which explains why citrulline was also significantly elevated in the TAC group [35]. The presence of multiple uremic toxin-related metabolites in whole blood samples suggests that the pathophysiology of kidney damage caused by TAC is reflected in the blood. These metabolites could become new indicators for TAC nephrotoxicity, which previously could only be assessed through kidney biopsies.

### 4.3. Metabolites Related to Vascular Disorders

Metabolites related to vascular damage and collagen degradation products were significantly higher in the TAC group. These metabolic changes show the detailed mechanism of specific arteriolar hyalinosis in kidney graft tissue of TAC nephrotoxicity [3,4]. Elevated levels of ADMA and SDMA are considered risk factors for progression of atherosclerosis and cardiovascular events [35,38], indicating the presence of vascular damage. γ-carboxyglutamic acid is a vitamin K-dependent amino acid synthesized in vascular smooth muscle and is involved in tissue anti-calcification [39,40]. On the other hand, γ-carboxyglutamic acid, which constitutes Matrix Gla Protein (MGP), accumulates at vascular calcification sites [40], and MGP levels are higher in the blood of patients with CKD and diabetes [41]. This has been suggested as a negative feedback response to vascular wall damage [42,43]. In this study, γ-carboxyglutamic acid was significantly higher in the TAC group, and it was previously observed in kidney samples from TAC nephrotoxicity. This suggests the presence of vascular damage in kidney tissue and that γ-carboxyglutamic acid may serve as a potential biomarker and that it contributes to TAC nephrotoxicity. Additionally, 6-aminohexanoic acid, which has anti-fibrinolytic effects, promotes coagulation and thrombus formation [44]. Coagulation is exacerbated by vascular damage, and high levels of 6-aminohexanoic acid in the TAC group may indicate hypercoagulation caused by vascular damage. As elevated levels of 5′-Methylcytidine have been associated with atherosclerosis, a higher ratio of 5′-Methlcytidine in the TAC group also suggests vascular damage caused by TAC [45,46].

Hydroxyproline, Pro-Hyp, 5-Hydroxylysine, and Gly-Pro-Hyp were also significantly elevated in the TAC group. These metabolites are products of post-translational modifications and components of collagens [47,48,49,50]. In clinical situations related to kidney transplantation, there is a characteristic pathological finding in renal tissue with hyaline arteriolar thickening called the ‘aah’ score [51,52]. This pathological finding indicates hyaline arteriolar thickening replacement of degenerated smooth muscle cells mainly due to long exposure to TAC or cyclosporine [51]. Since previous reports have described the main targets for TAC nephrotoxicity as vascular endothelial cells and arteriolar myocytes [3,6], TAC likely causes vascular injury. As vascular walls are collagen-rich structures, more abundant collagen peptides in blood samples in the TAC group indicate collagen leakage from vessel walls due to vascular injury by TAC.

### 4.4. Low NAD^+^ Levels and Salvage Pathway Impairment

NAD^+^ levels were significantly lower in the TAC group. NAD^+^ is an essential cofactor for energy metabolism, in which it functions as a coenzyme, and its systemic depletion is suspected to cause cellular senescence, metabolic disorders, especially in mitochondria, and immune dysfunction [53]. Focusing on vascular events, low NAD^+^ levels promote cellular senescence of vascular smooth muscle and vessel wall calcification [54,55]. Additionally, NAD^+^-dependent sirtuins are believed to help prevent vascular calcification and vascular smooth muscle aging, suggesting that low NAD^+^ levels suppress sirtuin function, leading to vascular damage [55]. Low NAD^+^ levels are also observed during both acute and chronic kidney injury [56].

On the other hand, mechanisms of NAD^+^ deficiency in vivo differ among diseases. Maintenance of NAD^+^ levels relies on a balance between biosynthesis and consumption. There are three biosynthetic pathways for NAD^+^: De-novo Biosynthesis, the Preiss–Handler Pathway, and the Salvage Pathway [56]. Among these, the NAD^+^ salvage pathway is primarily responsible for maintaining NAD^+^ levels [57]. Representative pathways that consume NAD^+^ include glycolysis, the citric acid cycle, poly (ADP-ribose) polymerases (PARPs), cADP-ribosyl synthetases, and sirtuins. NAD^+^ is converted to NADH in glycolysis and the citric acid cycle. In this study, quinolinic acid, a precursor of NAD^+^, was significantly lower in the TAC group. This suggests that the supply of precursors for NAD^+^ synthesis may be insufficient. On the other hand, NADH did not show significant differences between the two groups. This indicates that NAD^+^ deficiency was not mainly due to consumption in glycolysis or the citric acid cycle. In this study, NMN and NAM, metabolites related to the NAD^+^ salvage pathway [58,59], were also examined. While NMN showed no significant difference, NAM was higher in the TAC group. Additionally, ADP-ribose and NNO were significantly elevated in the TAC group.

ADP-ribose is a central metabolite generated by decomposition of NAD^+^, and elevated levels of ADP-ribose are associated with cellular senescence due to aging and various related diseases [60,61]. ADP-ribose is produced by consuming NAD^+^ through the PARPs and CD38. PARPs belong to a family known as ADP-ribosyl transferases and are promoted in response to DNA damage caused by oxidative stress, converting NAD^+^ into ADP-ribose and NAM [53]. NAD^+^ can also be broken down into ADP-ribose by CD38, which has both glycohydrolase and cADP-ribosyl synthetase activities [53].

NNO is an end product generated by oxidation of NAM [62,63]. This metabolite acts as an antagonist of C-X-C chemokine receptor 2 (CXCR2). Because CXCR2 is involved in renal inflammation and inflammation of peripheral blood vessels, NNO suppresses inflammation [62,63]. Elevated NNO levels in the TAC group suggest inflammatory responses in the vessels, potentially indicating compensatory increases.

From these findings, it can be concluded that the NAD^+^ deficiency in TAC nephrotoxicity results from a shortage of precursors for biosynthesis and a non-functional NAD^+^ salvage pathway. Impairment of the NAD^+^ salvage pathway is caused mainly by consumption of NAD^+^ and NAM utilized for production of NNO instead of NMN. This state of low NAD^+^ levels may be fundamental to the pathophysiology of vascular damage and CKD in TAC nephrotoxicity.

### 4.5. Metabolites Related to Cytosine and Its Aberrant Catabolites

In the current study, metabolites related to cytosine were significantly elevated. Ortic acid and 5′-methyl-2′-deoxycytidine increase in CKD [33,64]. DNA methylation is associated with CKD progression, pathology, prognosis, and renal fibrosis [65]. 5′-methyl-2′-deoxycytidine, one of the main DNA methylation markers, is thought to reflect pathophysiology of CKD [33]. In addition, Kesin et al. reported that 5′-methyl-2′-deoxycytidine is elevated in DNA damage [66]. Cytosine catabolism involves NAD^+^ as a coenzyme, and it is possible that restriction of cytosine degradation due to low NAD^+^ levels results in accumulation of cytosine-related metabolites and some DNA disorders in the TAC group.

### 4.6. Hypotheses on the Relationship Between NAD^+^ and TAC Nephrotoxicity

Previous studies have reported that TAC promotes production of ROS in blood vessels and kidney tissues, leading to tissue damage and fibrosis [24,25]. As ROS cause DNA damage, this may induce PARP activation, leading to NAD^+^ deficiency in the TAC group. In this study, metabolic changes associated with vascular and kidney damage were also observed. These phenomena were caused by NAD^+^ depletion due to impairment of the NAD^+^ salvage pathway. Impairment of the NAD^+^ salvage pathway in TAC-induced nephrotoxicity may contribute to vascular damage and CKD. Given that the pathological signature of TAC nephrotoxicity is specific arteriolar hyalinosis in kidney graft tissue, these metabolic changes, especially the relationship between vascular disorders and NAD^+^ depletion with the salvage pathway, offer more detailed insights into TAC pathophysiology and suggest avenues for further research.

The limitations of this study are as follows: This was an animal experiment using mice, so further research using human samples is required. Since blood samples were used, the observed metabolic changes may reflect not only renal damage, but also changes in other organs or the blood itself, including T-lymphocytes. In addition, the relationship between enzymes and substrates of each metabolite and their gene expression were not evaluated in this study. Although TAC nephrotoxicity was caused by prolonged exposure to TAC, these results only show metabolomic profiling and changes after 4 weeks of TAC administration and do not reveal the chronology of metabolic changes. However, we found many potential biomarkers for TAC nephrotoxicity and new keys to understand the pathophysiology of chronic damage caused by TAC. This study further clarifies the causes of severe problems induced by TAC nephrotoxicity for future development of treatments.

## 5. Conclusions

Metabolomic profiling of whole blood samples from mice with TAC-induced chronic nephrotoxicity was employed in this study. NAD^+^ depletion due to impairment of the NAD^+^ salvage pathway suggests that cellular senescence and mitochondrial dysfunction cause vascular disorders and CKD. Specific metabolites significantly different in both blood samples and kidney tissue were also revealed. These findings indicate that future investigations should focus on proteins and gene expression that change as a result of TAC chronic nephrotoxicity and that future research should examine the chronology of changes in affected metabolites to find biomarkers and therapeutic targets.

## Figures and Tables

**Table 1:** Metabolites in whole blood with significant difference between the two groups.

Metabolites	Ratio	*p*-Value	Significant Difference in Kidney Tissue *
threo-β-Methylaspartic acid	13	0.014	↑
γ-Carboxyglutamic acid	3.7	0.008	↑
5-Amino-3,4-dihydro-2H-pyrrole-2-carboxylic acid	3.4	0.019	
Ascorbate 2-sulfate	3.0	0.012	↑
Ribulose 1,5-diphosphate	2.6	0.045	
N-Carbamoylaspartic acid	2.3	0.022	
Cytidine diphosphate (CDP)	2.2	0.003	
N-Acetyllysine	2.2	0.044	
Glucaric acid	2.1	0.009	↑
Imidazole-4-acetic acid	2.1	0.035	
6-Aminohexanoic acid	1.9	0.034	
Orotic acid	1.9	0.015	
Nicotinamide N-oxide	1.9	0.014	
Adenosine diphosphate-ribose (ADP-ribose)	1.9	0.008	
N-Acetylgalactosamine 4-sulfate	1.8	0.005	
Deoxyadenosine diphosphate (dADP)	1.8	0.047	
Gamma-Aminobutyric acid (GABA)	1.8	0.016	
7-Methylguanine	1.8	0.045	
1-Methyl-4-imidazoleacetic acid	1.7	0.013	↑
β-alanine (β-Ala)	1.7	0.018	
2-Hydroxyisobutyric acid	1.7	0.006	
Imidazolelactic acid	1.7	0.003	↑
Glycine-Proline-Hydroxyproline (Gly-Pro-Hyp)	1.7	0.036	
Ascorbate 2-phosphate	1.7	0.017	
Allantoic acid	1.7	0.014	
5-Methyl-2′-deoxycytidine	1.7	0.016	
N-Acetylalanine-1 ^#^ N-Acetyl-β-alanine-1 ^#^	1.7	0.026	
N-Acetyltaurine	1.7	0.009	↑
Hydroxyproline	1.6	0.005	
Symmetric dimethylarginine (SDMA)	1.6	0.009	
2′-Deoxycytidine	1.5	0.005	↑
5-Hydroxylysine	1.5	0.026	
Isonicotinamide ^#^ Nicotinamide ^#^	1.5	0.032	
5-Methylcytidine	1.5	0.009	
N-Acetylgalactosamine ^#^ N-Acetylglucosamine ^#^ N-Acetylmannosamine ^#^	1.5	0.046	
Proline-Hydroxyproline (Pro-Hyp)	1.5	0.024	
Guanosine diphosphate-fucose (GDP-fucose)	1.5	0.012	
2-Deoxyribonic acid-1	1.5	0.043	
Phloroglucinol	1.5	0.023	
Asymmetric dimethylarginine (ADMA)	1.4	0.034	
myo-Inositol 2-phosphate	1.4	0.025	
Citrulline	1.4	0.012	
Pantothenic acid	1.4	0.013	
N, N-Dimethylglycine (DMG)	1.4	0.045	
N, N-Dimethylglycine	1.4	0.029	
Isocitric acid	1.3	0.035	
2-Hydroxy-4-methylvaleric acid ^#^ 2-Hydroxy-3-methylvaleric acid ^#^	1.3	0.046	
Creatinine	1.3	0.047	
Uridine diphosphate-galactose ^#^ (UDP-galactose) Uridine diphosphate-glucose ^#^ (UDP-glucose)	1.3	0.029	
Uridine diphosphate-N-acetylglucosamine ^#^ (UDP-N-acetylglucosamine) Uridine diphosphate-N-acetylgalactosamine ^#^ (UDP-N-acetylglucosamine)	1.3	0.038	↓
H-Aspartic (Glycine-OH)-OH (H-Asp (Gly-OH)-OH)	1.3	0.021	↑
Arabinonic acid-2	1.2	0.012	
Creatine	0.8	0.042	↓
Hercynine	0.5	0.034	
Quinolinic acid	0.4	0.048	
Nicotinamide adenine dinucleotide^+^ (NAD^+^)	0.3	0.024	

Ratio = normalized ratio of TAC group/normalized ratio of control group. ^#^: Detected possible metabolites, but not specified; *: Referring to a previous report; ↑: Significantly higher in TAC group; ↓: Significantly lower in TAC group.

## Data Availability

Datasets generated and/or analyzed during the present study are available from the corresponding author upon request.

## Supplementary Materials

The following supporting information can be downloaded at: https://www.mdpi.com/article/10.3390/antiox14010062/s1.
